# Why parents are skeptical about using probiotics preventively for small children: a Danish qualitative study

**DOI:** 10.1186/s12906-018-2387-2

**Published:** 2018-12-17

**Authors:** S. S. Andersen, K. F. Michaelsen, R. P. Laursen, L. Holm

**Affiliations:** 1 Department of Food and Resource Economics, University of Copenhagen, Rolighedsvej 25, 1958 Frederiksberg C, Denmark; 2 Department of Nutrition, Exercise and Sports, University of Copenhagen, Rolighedsvej 30, 1958 Frederiksberg C, Denmark

**Keywords:** Naturalness, Parents, Health perceptions, Probiotics, Small children, Qualitative

## Abstract

**Background:**

Research on the health effects of probiotics continues to grow, but less is known about consumers’ perceptions of probiotic products and their health effects, and the impact of these perceptions on consumption. Particularly little is known about the way parents perceive probiotic consumption by small children, and whether parental willingness to use probiotics as a treatment differs from their willingness to use them preventively. The aim of this study was to explore how parents perceive probiotic consumption by their small children, and their willingness to use such products in treatment and prevention.

**Methods:**

Semi-structured qualitative interviews with 17 Danish parents with at least one child aged 8–18 months. The interview guide centered on parental consumer practices and health-related attitudes both in general and in relation to probiotics. The data were coded in Nvivo and analyzed in a four-step analytical approach.

**Results:**

Parents are willing to use probiotics as a treatment but are skeptical about preventive use. Some parents define probiotics as a kind of medicine they use only if their child is ill. Probiotics also conflict with parental understandings of their children as small, perfect parts of nature. Parents worry that probiotics may cause an imbalance in the vulnerable perfection of a small child.

**Conclusion:**

The study shows that parental probiotic consumption practices are embedded in a cultural understanding of the child as both a perfect example of nature and vulnerable. Health authorities need to take this understanding into account if parents are to be successfully encouraged to use probiotics preventively.

**Electronic supplementary material:**

The online version of this article (10.1186/s12906-018-2387-2) contains supplementary material, which is available to authorized users.

## Background

Probiotics are defined by the Food and Agriculture Organization of the United Nations and World Health Organization [1] as “live microorganisms which when administered in adequate amounts confer a health benefit to the host” [1, 2]. Their potential health effects have attracted considerable research attention over recent years. They have been shown, for example, to reduce the incidence of severe necrotizing enterocolitis in preterm infants [3, 4], to be useful in the treatment of acute gastroenteritis [5, 6] and to reduce symptoms of atopic dermatitis [7]. Young children suffer from many infections, especially when they begin day-care [8, 9], and in Denmark 94% of infants aged 1–2 years attend day-care. Infections obviously have an impact on children’s wellbeing, but they also result in parental leave from work in dual-working families. This may result in pressure and reduced quality of life for these families. It also places a financial burden on society. Therefore, for a variety of reasons strategies to reduce the prevalence of infections in young children are needed. Probiotics might offer one such strategy.

This study is carried out as part of a larger project which investigated the effect of a combination of probiotics on young children’s absence from child care through a randomized, double-blind, placebo-controlled intervention [10]. While randomized intervention studies are important for documenting health effects of probiotics, they do not tell us whether it is possible to achieve these outcomes in real life [11]. For this to happen, parents must be willing to purchase probiotics and administer them in their children’s everyday diets. Therefore, the project also included a study with the aim to understand parents’ willingness to apply (and buy) probiotics in an everyday context and whether this would apply both to using probiotics as treatment and as prevention of disease. The study was based on in-depth qualitative interviews with parents and explored parental perceptions of probiotics for small children, and how these perceptions shape parental willingness to use probiotics for their children, either for treatment or prevention of disease.

We lack general knowledge about how consumers perceive probiotics, but it has been suggested that the market of probiotics lacks consumer confidence due to limited scientific evidence and lack of proper quality control resulting in too many ineffective products [12]. Even less is known about how specific consumer populations perceive probiotics. Thus, knowledge is missing about how parents perceive probiotics and the reasons they might have for choosing or declining to use probiotics for their children. A quantitative study has demonstrated that mothers have a high level of knowledge about what probiotics are, but the very same mothers were also found to be unsure about the health benefits of probiotics; one in three expressed uncertainty about whether it is safe to provide probiotics to their infants [13]. Currently, we do not know what explains this uncertainty, and it is therefore also unclear how best to respond to it. Parental willingness to provide their children with probiotics may, for example, depend on whether they interpret the purpose of the probiotics as preventive or therapeutic, on their interpretation of the product itself, or on their health beliefs in general [14].

Probiotic products can be administered as independent supplements (powder, tablets or capsules) or consumed in foods such as yoghurts. Both types of product were discussed with the parents.

## Methods

Before conducting the study, we had no information on how parents perceive probiotics. We therefore applied an explorative research design using a qualitative approach. Semi-structured interviews are a useful technique for gaining deep insight into respondents’ perceptions and the underlying reasons for those perceptions. They also allow interviewees to use their own words when describing their thoughts [15, 16].

Qualitative methods aim to analyze diversity in-depth rather than producing generalizable data which allow variation to be quantified. The aim was to gain access to, and explore, the mechanisms behind perception-processes affecting parental use of probiotics, not to measure the frequency or distribution of attitudes or practices. We therefore adopted a maximum variation sampling strategy [17], which is a purposive sampling technique where you include cases that are as different as possible. We recruited participants of either sex with at least one child aged 8–18 months. The participants were selected to represent various localities in Denmark and a range of sociodemographic backgrounds. The size of our sample is based on the principle of saturation [18] which is standard in qualitative research. It implies that researchers continue to gather data until new insights and new themes no longer emerge. In our case we reached the point of saturation after interviewing 17 parents. All received a gift card of approximately 40 EURO for a grocery shop as a token of gratitude.

We used a mixed recruitment technique including both network and snowballing strategies. The first author contacted four people in her network (gatekeepers) living in different geographical areas of Denmark. These people were invited to contact two or more further people who met the recruitment criteria and lived in their geographical area. These interviewees were then asked to contact further potential participants until enough data was gathered [17]. All of the interviews were carried out by the first author and took place in the homes of the interviewees. All interviews lasted about an hour.

The interview guide was designed to give insights into the consumption practices parents adopt when they have young children; parents’ health-related attitudes; the information sources parents trust when they seek health guidance; and parental thoughts on dietary supplements and probiotics. The guide was chronological. We first discussed health-related practices during pregnancy. We then moved on to experiences related to the child’s birth and the first months following that. When we reached the point at which we planned to discuss dietary supplements and probiotics, we began by talking about the interviewees’ experiences with probiotics and their thoughts about feeding them to their children. We then presented interviewees with a variety of products containing probiotics (yoghurt, drops and powder) and prompted them to reflect on the pros and cons of the different types of product (see a full version of the interview guide in Additional file 1).

All interviews were transcribed verbatim. This resulted in more than 600 pages of data. The interviews were analyzed in a four-stage process (see Mitchell & McClean [19] for a similar approach to data analysis). The first stage involved listening to the interviews in their entirety to understand interviewees’ individual stories. The second stage involved reading through the interviews and identifying salient themes within and across them. At the third stage we coded the data according to these themes and subthemes using the software Nvivo. And at the fourth stage we wrote a summary of each interviewee’s perspective on the main topics addressed in this paper. These topics included: how interviewees perceived a healthy childhood, what sources of information and advice they trusted in relation to health, their consumption practices, how they perceived probiotics, and what motivated them to use probiotics (or not to do so). We did this in an attempt to get a firmer hold on individual stories, but also to evaluate common themes that emerged across the interviews.

In total, 17 parents of small children aged 8–18 months participated in the study. The sample included 12 mothers and 5 fathers, 4 parents with higher education, 9 with a Bachelor’s or equivalent, 3 with vocational education, and 1 with elementary schooling. The parents were aged between 28 and 44 years. The participants lived in various localities all over Denmark including urban and rural areas. Five were resident in Denmark’s two largest cities (the capital area of Copenhagen and Aarhus) and 10 lived in smaller towns and rural areas. All parents were co-habitants and all were of Danish origin (i.e. born and raised in Denmark). The sample included parents with different levels of experience with the use of probiotics: 5 had never heard of probiotics before; 5 had heard of probiotics or lactic bacteria but never used them; 5 had used probiotics to treat their children’s stomach aches; and 1 couple had used probiotics as a means of prevention. To protect the identities of the interviewees, all participants are anonymized in this paper. Some of the data from this article is also analyzed in another study by Andersen & Holm about parental consumption routines and the management of risk [20].

## Results

Three major findings emerge from the four-stage analytical process we followed: a) naturalness appears to be a guiding principle in parental choices about what their children consume in general; b) parents are skeptical about the pharmaceutical industry, although they also accept that it promotes beneficial health effects; c) parents perceive probiotics as both natural and unnatural, and this duality makes it hard for them to evaluate the risks associated with probiotics.

### Naturalness as a guiding principle in a world of risky products

Many of the interviewees resisted probiotics for their children. This resistance was connected with their perceptions of healthy childhood and the way the product fitted into these perceptions. IP1 is a mother of two. She explains why, over a short period, she gave probiotics to her youngest as treatment, but would not consider using it preventively:

IP1: We did this (used probiotics) only because she had stomach ache. Otherwise I would never give my child Lactic acid bacteria if I didn’t think it was absolutely necessary.

Interviewer: No, why not?

IP1: Because I think we enter this world as small, perfect creatures, and we have the composition we have, and unless something is not right we should be able to function just fine. (From Andersen & Holm [20])

The interviewees described their newborn children as perfect parts of nature and indicated that since they are perfect there is no need to interfere with them. Feeding probiotics and similar products to infants is therefore not considered an everyday routine; rather it is something that parents like IP1 do (when they do) because they feel there is no alternative. IP1 did give her daughter probiotics, but only because she was advised to do so at a time when she described herself as desperate: *As parents we were simply desperate. Now, it [daughter’s diarrhea] had to stop, right? She pooped all the way up to her neck. So then you try to do stuff [like providing probiotics] that can bring back a balance again, right?*

Overall the interviews suggest that parents perceive various consumption practices chosen for their children as a risk-related practices, and that they apply the concept of naturalness as part of a risk reduction strategy. For example, IP2, a mother of two girls, reported that she always reads product labels carefully, with everything from car seats to food, in order to avoid what she characterizes as artificial, harmful ingredients:

IP2: Yes, I always read product labels.

Interviewer: Yes, why?

IP2: Because there is aspartame in so many things. And I don’t like the thought of artificial sweeteners, for example. So therefore I read [labels], because sometimes it says ‘light’ or ‘no added sugar’, but whenever it says ‘no sugar’ then there is probably something else in it. And I prefer the natural unhealthy stuff rather than the artificially produced unhealthy stuff.

Like IP2, other interviewees applied naturalness as a guiding principle when explaining their reasons for buying various things, from organic food products to things made from natural sources such as toys made of natural rubber, leather shoes, or lambskin. It was apparent that they took such products to contain fewer harmful, artificial additives and ingredients. In the following remarks IP3, a mother of two, explains why she refuses to use skincare for her children:

IP3: I don’t think that covering your kids in lotion and stuff… I can’t see any reason to do that, it doesn’t seem natural. If I had to use lotion, they [the kids] should suffer from a particular dry skin or something like that. But I haven’t experienced that.

Interviewer: What do you mean by natural?

IP3: Well, well… [pause]. What can you say? I don’t think… [pause]. The natural thing is to do nothing. That is how it was originally. But today there are all these soap products, and then you have to be careful because they can cause an imbalance in the natural fat balance in your skin. (From Andersen & Holm [20])

IP3’s comments point up several important issues. First, it can be seen that she understands skincare products for children as unnatural. Second, in her view doing nothing and letting nature go untouched is the natural thing to do. And third, it emerges that she assumes the ‘natural’ thing to do is the right thing to do. Products may do more harm than good, according to this logic. And children are to be protected from the world of unnecessary products, because children are parts of original nature just as they are, and to interfere with untouched nature is to risk contamination. Too much interference from unnatural products might cause an imbalance in the part of nature the child represents. So, naturalness is applied by parents because it is seen as an efficient tool for managing consumption choices. It eliminates many choices (e.g. products which are not perceived as natural) while shielding children from artificial additives and ingredients that are (perceived to be) harmful.

### Skepticism about the industry

IP4, a dad of a son, adds another dimension to the explanation of why products like probiotics do not fit in the parental story of nature and the newborn child (See also Andersen & Holm [20]):

Then there is this little thing that nature does not have to earn money. Those who produce, for example, Lactocare [a supplement with added probiotics] have to [do that].

Nature is to be trusted. Unlike products produced by the pharmaceutical industry, including probiotics, it does not care about profit margins. IP4 considers pharmaceutical companies as too profit-oriented, and perceives a risk that such companies might neglect potentially damaging side-effects or provide useless products simply in order to generate profits. The description of nature as unquestionably good contrasts sharply with the way IP4 characterizes the pharmaceutical industry as greedy. However, it seems that it is not just the pharmaceutical industry that parents distrust, but the commercial sector in general, since a similar description emerged when some of the interviewees talked about milk. Some parents argued that it is not natural for humans to drink cow’s milk. They also said that the milk industry in Denmark is too involved in developing the dietary guidelines for children. They basically distrusted the guidelines, and they tended to serve their children less cow’s milk than the Danish guidelines recommend. Although only few of the interviewees questioned the milk industry, we can see how nature was once again used as a tool enabling parents to navigate in a world of consumption.

Nature had an almost religious status in the minds of the interviewees, and although it is well known that nature can often be both damaging and harmful, it seems the parents felt that nature is more reliable than either science or the food and health industries. This reveals an interesting tension: whereas the purpose of probiotics is to reduce risk, the reason they are avoided is because they are connected, in parents’ minds, with risks.

### Interviewees’ definitions of probiotics

Different definitions of probiotics transpired in the qualitative interviews, suggesting that probiotics are perceived as a rather complex product. Many interviewees associated the use of probiotics with some sort of medical treatment, regardless of whether the probiotic was incorporated in a foodstuff such as yoghurt or accessed independently as powder or drops. A few parents perceived probiotics in food as a more natural way of consuming probiotics but due to the young age of their children they would not necessarily purchase probiotics in this shape. The majority of parents said that they would choose the product that was most feasible, i.e. the product that they could make their children consume most easily. So, although some parents might have perceived yoghurt as more natural, feasibility was a decisive criterion.

For a while, IP5’s daughter suffered from stomach ache, and on the advice of a health care practitioner she gave probiotics to her daughter during this period. IP5 defined probiotics as follows:

I: So what do you think it is [a probiotic]? Is it a kind of dietary supplement or…? Or what?

IP5: No, it… I think it is kind of like medicine. So it helps her get rid of her flatulence.

I: But why did you then stop feeding her probiotics? Is it because now it is working, then?

IP5: Well, she didn’t have… Actually yesterday was the first time she had [stomach ache]. The past two months there hasn’t been anything wrong, so that is why I stopped feeding her [probiotic supplements], because I thought: No, it is not necessary to use it, when she doesn’t have stomach ache anymore.

I: No. So there has to be some kind of a problem before you think it is necessary to use?

IP5: Yes.

IP5’s definition of probiotics as a medical product means that she (like other parents we interviewed) assumed the product only had to be used for treatment purposes, and thus did not think of probiotics as prophylactic. Like IP5, many of the interviewees therefore avoided using probiotics unless their child was ill, in which case any use should follow the recommendations of a trusted health authority. The parents explained that there are so many products, and there is such a lot of good advice ‘out there’, that they felt they would need some professional guidance on how to distinguish good products from bad ones.

Not all of the interviewees agreed that probiotics are a medicinal treatment. But the dissenters here had a hard time defining what probiotics are in that case. Some defined it as a dietary supplement, or as something lying between dietary supplements and medicine. IP6, a mother of two children, used probiotics for her youngest child. She was advised to do so by her zone therapist, as her son suffered from stomach aches: *I think that it [Lactic acid bacteria] is something … well it is something natural you need to have, right? Maybe it is a bit more unnatural when it comes like this [probiotics in drops], but this is the way we can get it.*

In these remarks, IP6 tries to come to a decision about the probiotic’s risk status by categorizing it according to its perceived naturalness. As described in the first section, naturalness is thus regarded as a quality that separates safe products from unsafe products. But it is not easy for IP6 to decide whether probiotics are natural. In this respect, she is like the other parents. On the one hand, they argue that the product is natural because lactic bacteria are microorganisms, which by nature are already in our bodies and in food. On the other hand, they perceive probiotics as something unnatural, because they think these products enters the body in an unnatural way.

## Discussion

The interviews reported above underline the fact that we must understand parental use of probiotics as a cultural phenomenon and not just as a question of parents weighing costs and benefits. Parental perceptions of probiotics are embedded in understandings of children as vulnerable and pure, in the idea that unnatural products are potentially harmful, and of course in the self-understanding that parents are responsible for managing risk through their consumption practices. The explorative qualitative approach applied in this study proves to be an important way to gain insight into this phenomenon. Thus, the study not only provides evidence that parents perceive probiotics ambivalently, but also reveals the underlying reasons *why* parents hesitate to use probiotics preventively. Parents regard probiotics as a product that is both natural and unnatural, a dual position which conflicts with parents’ understanding of their newborn child as a pure part of nature in danger of being contaminated by too much interference from unnatural products.

Overall, our findings suggest that there is a discrepancy between the way experts understand probiotics and the way the potential users of such products perceive them. Experts may document the preventive and beneficial effects of probiotics, and explain how the product can be used to boost a child’s immune system, but parents appear to continue to resist using the product preventively.

Children are increasingly considered ‘at risk’ in Western contemporary societies. Research has shown that parents are anxious about the health and safety of their children [21–23], and that their concerns relate, for example, to both the leisure activities their children participate in and the food they eat. Theoretically, this growing concern and close monitoring of our children are linked to Giddens’ [24] and Beck’s [25] idea of the rise of a risk society. Today we exploit many different sources of risk-information, from social media to commercial advice, and often we encounter conflicting messages even from experts. Within this myriad of information individuals have to decide who they will trust. In doing so, they adopt a reflexive mode to reduce risks, and individuals constantly calculate the pros and cons of different behaviors [26]. Consumption patterns involving children are no exception. In fact, reflexive conduct seems to be reinforced as a consequence of the vulnerable state of small children: parents feel they are navigating their way through a world of risks. One way of dealing with the anxieties that may arise from this – and a policy that is clearly visible in the results presented in this study – is by applying naturalness as a guiding principle. The naturalness principle can be seen as an example of what Nichter and Thompson refer to as a “harm reduction strategy” [26]. Such a strategy is applied by individuals when they face an interplay between a growing awareness of their exposure to risks, a vulnerability to illness, and an increasing feeling of personal responsibility for their own health [26]. In the present case, the personal responsibility, felt by the parent, extends to the health of the child, and, as has been described, feelings of vulnerability and responsibility may be reinforced in a parent-child relationship [20].

The implications of these findings are several. To begin with, if parents are to be successfully encouraged to use probiotics preventively, health authorities will need to find ways to remove parental misinterpretations of what probiotics are. One way to do this would be through renewed communication strategies. Our results suggest that it is important that the messages come from health authorities, and not directly from the probiotics industry, because this industry does not enjoy parental trust. The messages should build on specifically parental understandings of the infant, and consequently they need to stress that probiotics do not cause an imbalance in the natural development of small children.

It is, however, important to acknowledge that this study is not generalizable to the whole population: a rather small study sample was recruited, and single-parent households and ethnic minorities were not represented in that sample. This is a limitation of the study: single parents may be more willing to use probiotics because absences from work made unavoidable by a child’s illness may create a bigger problem for them than they do for parents who share childcare responsibilities with a partner. Equally, ethnic minorities may apply understandings of health, illness and nature that differ from those of the parents we have interviewed. Future studies on this subject should therefore prioritize the inclusion of various types of family and ideally interview larger sample groups.

In sum, our findings have implications for the way in which health authorities communicate new health-promoting initiatives relying on probiotics and similar products. If parents are to offer their children probiotics as a preventive measure, health authorities will need to provide more information and guidance about what probiotics are and how they benefit children.

## Conclusion

Many parents, though not all, prefer not to use probiotics preventively. They do not consider feeding dietary supplements to their children unless advised so by their general practitioner or another trusted health authority. Some parents are willing to use probiotic products as a treatment if their child has a condition that could be alleviated using such a product. But again, they typically consult a trusted health authority before going ahead. Given that probiotics can be used as a preventive measure, the parental definition of the product as a therapeutic medicine is arguably a lost opportunity. The definition reveals that perceptions in parental consumption patterns involving children are highly complex. This cannot be ignored by researchers if the preventive benefits of probiotics for children’s health are to be secured.

## Additional files

Additional file 1: Interview guide. Semi-structured interview guide for interviews with parents of young children. (DOCX 20 kb)
