# Supplementary material for: Why parents are skeptical about using probiotics preventively for small children: a Danish qualitative study
Source: BMC Complement Altern Med. 2018 Dec 17;18:336. doi: 10.1186/s12906-018-2387-2 (PMC6296026; doi:10.1186/s12906-018-2387-2)
Supplement: Supplementary file 1 — Interview guide. Semi-structured interview guide for interviews with parents of young children. (DOCX 20 kb) [file 12906_2018_2387_MOESM1_ESM.docx]

## Interview guide with parents of young children

| **Theme** | **Question** |
| --- | --- |
| Introduction | Inform about the purpose of the interview  Inform about anonymity |
| Background information | Age  Education  Job  Number of children, age of children  Civil status  Income/household income  Illness in the family |
| Pregnancy | How did the pregnancy go? (if several children, was it then different from the first pregnancy)  During pregnancy, did you think about your child's health, well-being, for example in relation to:  - what you ate  - exercise  - clothes  - make up  - dietary supplements  - medicine  - flu vaccine  -alcohol  -smoking  [If dad: did you discuss these issues with the mother of your child]  Did you experience situations during pregnancy where you had doubts if your child was during well? What did you do? Why?  Did you experience situations where you doubted if you made the right – healthy – choice? What did you do when you were uncertain?  Did you receive any guidance or advice about the health of your child during pregnancy  Did you seek information yourself for example from the Internet? Why?  What is a good pregnancy? |
| Birth | How did the birth go?  Mothers: Did you have any thoughts about how the birth should be carried out e.g. painkillers or not?  And what about after giving birth, did you stay at the hospital or did you go home? Why?  Did you experience any worries about the health of the baby after/during the birth?  What is a good birth? |
| First few months | Did you nurse? Why? When did you stop? Why did stop? [If dad – did the mother nurse]  How did it go with complementary feeding? Did you seek any information about dietary recommendations for this age? Did you read the guideline from the Danish health authorities about complementary feeding?  Did you feed him/her vitamin D?  Did you have a visit from the health nurse? How did you experience the visit?  Did your child follow the growth chart – and did you think about? What did this mean to you (that he/she did follow or did not follow the chart?  Do you follow the Danish vaccination guidelines? Did you have any thoughts about this?  Have you experienced situations where you felt you had conflicting health messages e.g. some experts contest the HPV vaccine, while authorities recommend girls to have the vaccine?  Only mothers: Did you join a mothers’ group? Did you discuss topics of health and well –being? Was that a comfort?  What is a healthy childhood? |
| Products/consumption and probiotics | Have you thought about the health and safety of your child in relation to consumption of products e.g.  Organic products  Lotion and soap products  Baby slings  Clothes  Car  Indoor climate e.g. wood burner  Toys  Car safety seat  Convenience food  Antibiotics  Have you sought information about products? Do you read e.g. product labels?  Do you know about probiotics?  What did you think about probiotics the first time you heard about it?  How did you hear about it? Who introduced it to you?  Have you used it for your child? Why/why not?  Would you use it? Why/why not?  [Interviewer then introduces 3 different Probiotic products: powder, yoghurt and drops]  Which of these products would you prefer feeding your child? Why?  Do you and the mum/dad agree about what products are healthy or not? |
| Caring practices | Do you think about the health and safety of your child during the day e.g. when he/she eats, plays, sleeps ect.  Who do you turn to if any – when you experience concerns about your child’s health?  Have you experienced feeling too protective or maybe the opposite? |
| Reflections about parenting | Did anything surprise you about becomming a parent?  Are there things that you did different from your first child to now?  Have you experienced being in a situation where you had doubts about making the right healthy decision on behalf of your child? |
